# Ginsenoside Rg1 promotes astrocyte‐to‐neuron transdifferentiation in rat and its possible mechanism

**DOI:** 10.1111/cns.14000

**Published:** 2022-11-09

**Authors:** Kelv Shen, Duanrong Wu, Baihan Sun, Yin Zhu, Hao Wang, Wenjun Zou, Yuhang Ma, Zhengfeng Lu

**Affiliations:** ^1^ Department of Orthopedics The Second Affliated Hospital of Soochow University Suzhou China

**Keywords:** ginsenoside Rg1, neuron, reactive astrocytes, regeneration, spinal cord injury, transdifferentiation

## Abstract

**Introduction:**

Neuronal loss caused by spinal cord injury (SCI) usually contributes to irreversible motor dysfunction. Promoting neuronal regeneration and functional recovery is vital to the repair of SCI.

**Aims:**

Astrocytes, activated by SCI with high proliferative capacity and proximity to neuronal lineage, are considered ideal cells for neuronal regeneration. As previous studies identified several small molecules for the induction of astrocyte‐to‐neuron, we confirmed that ginsenoside Rg1, a neuroprotective herb, could promote the direct transdifferentiation of astrocyte‐to‐neuron in rat.

**Methods and Results:**

Immunofluorescence staining showed that 26.0 ± 1.5% of the induced cells exhibited less astroglial properties and more neuronal markers with typical neuronal morphologies, reflecting 20.6 ± 0.9% of cholinergic neurons and 22.3 ± 1.9% of dopaminergic neurons. Western blot and qRT‐PCR revealed that the induced cells had better antiapoptotic ability and Rg1‐promoted neuronal transdifferentiation of reactive astrocytes might take effect through suppressing Notch/Stat3 signal pathway. In vivo, a revised SCI model treated by Rg1 was confirmed with faster functional recovery and less injury lesion cavity.

**Conclusion:**

In summary, our study provided a novel strategy of direct transdifferentiation of endogenous rat reactive astrocytes into neurons with Rg1 and promotion of neuronal regeneration after SCI.

## Graphical abstract

Ginsenoside Rg1, a neuroprotective herb, could promote the repair of injury neurons and reverse motor dysfunction caused by spinal cord injury (SCI). In this study, ginsenoside Rg1 is proven to have positive effects on the directly transdifferentiation of astrocyte‐to‐neuron after SCI in vivo and vitro. The underlying mechanism of this process may proceed by blocking Notch/Stat3 signal pathway. The intervention of ginsenoside Rg1 could give a novel and optional select to the clinical applications.

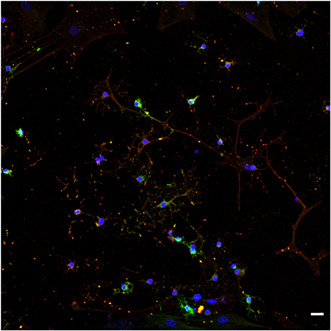

## 1 INTRODUCTION

Spinal cord injury (SCI) is a severe incidence that contributes to deformity or even mortality, causing a big burden both on families and society.^1^ A series of microenvironment changes in the epicenter of lesion after SCI inhibit the repair of neurons and finally hinder the motor functional recovery.^2^ Following directly instantaneous impact after SCI, the secondary injury caused by inflammation and gliar scar formation is more fatal and complex leading to permanent neuronal loss at the injury lesion site.^3^

Astrocytes, playing the major part of role in the central nerve system (CNS), serve as the providers of nutrition and supports which help neurons through promoting synapse formation, shaping synapse, and maintaining related homeostasis.^4^ According to major previous views, however, astrocytes would be activated to proliferate into gliar scars, mainly resulting in the obstruction of neuronal regeneration.^4^, ^5^ Nevertheless, some recent studies showed that astrocytes and gliar scars also have a double‐aged effect of both neuroprotective functions and neuroinhibitory functions during the process of SCI.^6^, ^7^

Neuronal transdifferentiation has become a promising therapy that aims at repairing SCI in recent years.^8^, ^9^ Relying on viral‐based expression of transcription factors, somatic cells transdifferentiation used to be one available approach, the other one might be stem cells transplantation.^10^, ^11^ However, the potential risks of tumorigenesis and the difficultly in delivering exogenous genes in vivo remain unsolved during their clinical applications.^12^, ^13^ Small‐molecule‐mediated chemical transdifferetiation has also received much attention. Not like the two approaches above, small chemical molecules are more controllable and safer when they come to therapeutic applications.^14^, ^15^

Panax ginseng, serving as a traditional Chinese herb, has been used medically for thousands of years. Ginsenoside Rg1 is one of the main active ingredients of panax ginseng.^16^ It has been widely confirmed that ginsenoside Rg1 plays an important part in the protection of cardiovascular system, immune system, and nervous system.^17^, ^18^ In addition, more and more evidences have been proved that ginsenoside Rg1 is conducive to the repair of injury neurons, the suppression neuronal apoptosis, and the regeneration of neuronal axons.^19^, ^20^

In the present study, we conducted a novel approach that utilizing ginsenoside Rg1 could directly convert reactive astrocytes (RAs), activated by TGF‐β1, into neuron‐like cells (iNs) in vitro. Interestingly, the whole transdifferentiation did not go through the middle stage of neuronal proliferation. Meanwhile, in vivo experiments, ginsenoside Rg1 promoted the repair of rats' motor function and reduced the region of lesion in spinal cord. This different therapy may become a promising select when facing neuronal degeneration after SCI. Furthermore, the RA‐to‐neuron transdifferentiation process influenced by ginsenoside Rg1 maybe take effect by blocking the Notch/Stat3 signal pathway.

## 2 MATERIALS AND METHODS

### 2.1 Isolation and culture of primary RAs

All the experimental procedures used in this study were examined and approved by the Committee of Animal Care and Ethics at the Animal Facility of Soochow University, Jiangsu, China. All efforts were made to minimize the number of animals used in this study and their suffering.

The Sprague–Dawley (SD) rats were purchased from Zhaoyan Co. Primary astrocytes were isolated from the brains of 1–3‐day‐old neonatal SD rats according to the previous studies. The protocol had been improved slightly.^20^ Briefly, the rat pups (male and female) were decapitated with sterilized instruments following painless sacrifice. After careful removal of medulla, brain stem, pia mater, and vessels under a dissecting microscope, the remaining cerebral cortex were minced and maintained in the Dulbecco's modified Eagles medium (DMEM)/F12 (Gibco, 11320033). The whole process was conducted on an ice box. After the digestion of 0.125% trypsin–EDTA solution (Beyotime, C0201) for 20–30 min at 37°C, DMEM/F12 supplemented with 10% fetal bovine serum (FBS, Gibco, 10099141) was added to terminate the process. The suspension of digested tissue was filtered through a nylon mesh (pore size, 74 μm) and then precipitated by centrifugation at 258 × *g* for 5 min. The final single‐cell suspension was cultured in T25 flasks at 37°C in a humidified 5% CO_2_, 95% air atmosphere for 7 days to obtain mixed glial cultures. Then, the mixed glial cultures were shaken at 240 rpm overnight to remove microglia and oligodendrocyte precursor cells (OPCs). After passaging to the third generation, 10 ng/ml TGF‐β1 (PeproTech, 100‐21) was added to obtain in vitro RAs at a density of 5 × 10^5^ cells/well in six‐well plates for 24 h. Thereafter, subsequent experiments were performed when glial fibrillary acid protein (GFAP) positive cells exceeded 95% of the total cell number.

### 2.2 Isolation and culture of primary rat neurons

The single‐cell suspension was obtained by the method described above and cultured in the neuron culture medium supplemented with neurobasal‐A medium (Gibco, A2477501), 2% B27 (Gibco, 17504044), 1% of 100× GlutaMAX (Gibco, 35050061), and 1% penicillin–streptomycin (Beyotime, C0222). Then, the culture medium was replaced at the 4, 45, and 96 h.Finally, high purity rat neurons were harvested on the 5th day.

### 2.3 Neuronal induction from RAs into iNs

After the process described previously, the culture medium was replaced with induction medium (IM) comprising neurobasal‐A medium supplemented with 0.5% N2 (Gibco, 17502001), 1% B27, and 1% penicillin–streptomycin as well as various concentration of ginsenoside Rg1 (Selleck, S3923) in the test group or same volume of dimethyl sulfoxide (DMSO) in the control (Ctrl) group. The induction medium plus ginsenoside Rg1 was replaced every 2 days. On the indicated days, the culture medium was replaced with neuron maturation medium (NM) comprising neurobasal‐A medium supplemented with 0.5% N2, 1% B27, 20 ng/ml brain‐derived neurotrophic factor (BDNF, Peprotech, 450‐02), 20 ng/ml glial cell‐derived neurotrophic factor (GDNF, Peprotech, 450‐10), 10 ng/ml NT3 (Peprotech, 450‐03), and 10 μM forskolin (Sigma, F3917). Half of the culture medium was replaced with fresh medium every other day until the indicated days.

### 2.4 Edu incorporation assay

Twenty‐four hours prior to fixation, 10 μM Edu was added on the 0, 24, 48, and 72 h of induction. Cells were fixed by 4% paraformaldehyde for 20 min and permeabilized with 0.5% Triton X‐100 in phosphate‐buffered saline (PBS, Solarbio, P1010) at room temperature. The following Edu staining was performed according to the manufacturer's instructions of the Edu cell proliferation detection kit (RiboBio, C10310‐1). Thereafter, cells were detected with GFAP or microtubule‐associated protein 2 (MAP2) immunofluorescence staining. Images were captured and analyzed with ImageJ software.

### 2.5 Immunofluorescence staining

On the indicated day, all culture cells were fixed in 4% paraformaldehyde for 20 min and permeabilized with 0.3% Triton X‐100 in PBS for 15 min at room temperature. Subsequently, the cells were washed three times with PBS and then incubated with 5% bovine serum album (BSA) in PBS for 1 h. After that, the cells were incubated with primary antibodies at 4°C overnight. Then, the cells were washed three times with PBS and incubated with secondary antibodies in the dark for 1 h. Cell nuclei were stained with 4′,6‐diamidino‐2‐phenylindole (DAPI, Sigma, D9542). Images were captured using a laser scanning confocal microscopy (Zeiss) and analyzed with ImageJ software. The following antibodies were used: rabbit anti‐GFAP (1:500, Proteintech, 16825‐1‐AP), mouse anti‐GFAP (1:500, CST, 3670), mouse anti‐class III β‐tubulin 1 (TUJ1, 1:100, Abcam, ab78078), rabbit anti‐MAP2 (1:500, CST, 4542), rabbit antineuronal nuclei (NeuN, 1:500, CST, 12943), rabbit anti‐PAX6 (1:500, Proteintech, 12323‐1‐AP), rabbit anti‐NESTIN (1:100, Proteintech, 19483‐1‐AP), rabbit anti‐SOX2 (1:500, Proteintech, 11064‐1‐AP), rabbit anti‐choline acetyltransferase (CHAT, 1:100, Proteintech, 20747‐1‐AP), rabbit anti‐Ki67(1:250, Abcam, ab16667), rabbit antityrosine hydroxylase (TH, 1:500, Proteintech, 25859‐1‐AP), rabbit antivesicular glutamate transporter 1 (vGlut1, 1:500, Abcam, ab227805), rabbit anti‐synapsin‐1 (SYN1, 1:500, Abcam, ab254349), goat antirabbit Alexa Flour 488 (Abcam, ab150077), and goat antimouse Alexa Flour 594 (Abcam, ab150120).

The measurement of cell purity, cell proliferation, neuronal subtype, neuronal transdifferentiation efficiency, total neurite branch length, and total neuronal territory size were calculated as previously described with some minor modifications. Briefly, after the immunofluorescence staining of induced cells, five randomly selected 10× or 20× visual fields were used to count cell numbers. The cell purity was calculated by the ratio of the number of glial cells or neuronal cells to the total cell number indicated by DAPI. The cell proliferation rate and neuronal subtype were calculated similarly. The transdifferentiation efficiency was calculated by the ratio of the number of the number of MAP2‐positive cells to that of initial cells seeded in each field. As TUJ1 could be expressed in the cytosol of neurons, the neurite length was calculated by TUJ1 immunofluorescence staining from the cell body to the end of the neurite by ImageJ with NeuronJ plugin.

### 2.6 Western blot analysis

The induced cells or tissue from lesion area were scraped and lysed in radioimmunoprecipitation assay (RIPA) buffer (Beyotime, P0013) on ice for 1 h at 4°C. After the centrifugation of lysates at 18,407 x *g* for 15 min, the protein concentration of supernatant was measured by using a bicinchoninic acid kit (BCA assay kit, Beyotime, P0010) according to the manufacturer's instructions. The cell lysates were diluted 4:5 (v/v) in sodium dodecyl sulfate (SDS) sample buffer (Beyotime, P0015). Equal amounts of protein were separated by 10% or 15% SDS‐polyacrylamide gel electrophoresis (80 V for 90 min). Then, the protein was transferred to polyvinylidene difluoride (PVDF) membranes. Next, the membranes were blocked with 5% BSA in tris‐buffered saline containing tween 20 (TBST) for 1 h at room temperature followed by incubation overnight at 4°C with primary antibodies against complement component 3 (C3, 1:1000, Abcam, ab97462), Notch‐1 (1:1000, Proteintech, 10062‐2‐AP), Stat3 (1:1000, Proteintech, 10253‐2‐AP), p‐Stat3 (1:2000, CST, 9145), MAP2 (1:1000, CST, 4542), NeuN (1:1000, CST, 12943), GFAP (1:2000, Proteintech, 16825‐1‐AP), GAPDH (1:20000, Proteintech, 60004‐1‐Ig), Bax (1:5000, Proteintech, 60267‐1‐Ig), B‐cell lymphoma 2 (Bcl‐2, 1:1000, Proteintech, 26593‐1‐AP), and cleaved‐caspase‐3 (1:1000, CST, 9664). After washed three times with TBST, the membranes were incubated with horseradish peroxidase (HRP)‐conjugated secondary antibody (1:5000, CWbio, CW0102, CW0103) for 1 h at room temperature. Subsequently, protein bands were visualized with the BeyoECL Plus (Beyotime, P0018) detection system, and the intensities of them were analyzed via densitometric analysis using the ImageJ software.

### 2.7 Quantitative reverse transcription‐polymerase chain reaction (qRT‐PCR) analysis

Total RNA was extracted from the indicated samples using Trizol (Invitrogen) and was reverse transcribed by using a Maxima H Minus First Strand cDNA Synthesis Kit (ThermoFisher, K1651) following the manufacturer's instructions. Quantitative reverse transcription PCR involved the use of Novostart SYBR qPCR SuperMix Plus kit (Novoprotein, E096‐01B) in a One‐Step qRT‐PCR System (Bio‐Rad CFX96 Touch). Target gene expression was normalized to *GAPDH* expression (internal control) using the $2^{-∆∆C_{T}}$ method. All primer sequences were listed below:

*C3*: 5′‐GGAGATTCTGGCAGTGAGCTTGTC‐3′ (forward) and
5′‐GGGCAGTCGCAGGTCAATGAAG‐3′ (reverse);
*GFAP*: 5′‐GGTGGGCAGGTGAGGAAGAAATG‐3′ (forward) and
5′‐CTGAAGGTTAGCAGAGGTGACAAGG‐3′ (reverse);
*NEUROD1*: 5′‐GGAACACGAGGCAGACAAGAAGG‐3′ (forward) and
5′‐CATCCTCCTCTTCCTCCTCCTCTTC‐3′ (reverse);
*Myt1l*: 5′‐TCTGGACACATCACTGGCAATTACG‐3′ (forward) and
5′‐CTGATTGGCTCCTGGTCTTCCTTG‐3′ (reverse);
*TUJ1*: 5′‐CATGAAGGAGGTGGATGAGCAGATG‐3′ (forward) and
5′‐GTTGCCGATGAAGGTGGACGAC‐3′ (reverse);
*MAP2*: 5′‐CCAGAACATACCACCAGCCCTTTG‐3′ (forward) and
5′‐TCCTCTCGTCAGCCATCCTTCAG‐3′ (reverse);
*SYN1*: 5′‐GTCCTCATTCGTGCTGCCTGTG‐3′ (forward) and
5′‐AGGAGTGGAGGTTGGAGGAAGATG‐3′ (reverse);
*SMN1*: 5′‐AGTGGTGTCATCATCAGGGTCTCC‐3′ (forward) and
5′‐GTTCCTCCAACTGCTGCTCTATGC‐3′ (reverse);
*ISL1*: 5′‐ACCCTCTCAGTCCCTTGCATCC‐3′ (forward) and
5′‐GGTGGTCTTCTCGGGCTGTTTG‐3′ (reverse);
*CHAT*: 5′‐AGGAAGGAAGGAAGGAAGGAAGACC‐3′ (forward) and
5′‐TTCACCCACAACCCAGCTTTAGTTC‐3′ (reverse);
*Hey1*: 5′‐CGGGAATGCCTGGCTGAAGTTG‐3′ (forward) and
5′‐GGGATGCGTAGTTGTTGAGATGGG‐3′ (reverse);
*Hes1*: 5′‐CTAACGCAGTGTCGCCTTCCAG‐3′ (forward) and
5′‐AGAGAGGTGGGCTAGGGAGTTTATG‐3′ (reverse);
*Notch‐1*: 5′‐GCCAGCAAGAAGAAGCGGAGAG‐3′ (forward) and
5′‐CCACTCGTTCTGATTGTCGTCCATC‐3′ (reverse);
*GAPDH*: 5′‐GACATGCCGCCTGGAGAAAC‐3′ (forward) and
5′‐AGCCCAGGATGCCCTTTAGT‐3′ (reverse).

### 2.8 Preparation of rat SCI model

The SCI model was established in rats using Allen's weight dropping method as described previously.^21^ Briefly, 30 adult female SD rats (180–220 g) were anesthetized with 2% halothane and were performed a laminectomy to expose the spinal cord at the spinal T9‐T10 segments. Then, a weight‐drop impact was performed using a 10‐g Kirschner wire dropped from a height of 40 mm. Next, the muscles and skin were quickly sutured, and the rats were randomly divided into three groups: Rg1 treatment group, vehicle treatment group, and sham group (*n* = 10 for each group). After the surgery, the rats received an i.p. injection of 10 mg/ml ginsenoside Rg1 in water (10 ml/kg body weight) or vehicle solution (saline), daily for consecutive days. The sham group underwent the same surgical procedure without the impact of a weight‐drop. Each group was kept in separate cages with free access to food and water. In addition, all the bladders of rats were manually and gently voided twice per day until the urinary reflexive control of micturition was recovered.

### 2.9 Behavior analysis

The Basso‐Beattie‐Bresnahan (BBB) scale was used to assess functional recovery before surgery and on 1, 3, 7, 14, 21, and 28 days post injury. The assessment of this scale was based on the relative principles of previous studies.^21^ There were four parts of this assessment: joint activity (number and range), degree of weight bearing and coordination of movement, trunk stability during exercise, and activity of the tail. Two blinded and independent observations were performed by two observers who were not familiar with this experiment but familiar with the BBB scoring method. Each rat was observed for 4 min in the daytime. A final score of 0 indicated complete paralysis, and a final score of 21 indicated complete mobility.

### 2.10 Immunohistochemistry staining

The transverse or longitudinal paraffin sections of tissue were incubated in 3%H_2_O_2_ for 15 min and then immersed in blocking solution for 1 h at room temperature. After that, the sections were incubated with primary antibody (rabbit anti‐MAP2, 1:500, CST, 4542) at 4°C overnight. Then, the sections were washed three times with PBS and incubated with HRP‐conjugated secondary antibody at 37°C for 1 h. After stopped with 3,3‐diaminobenzidine (DAB, Sigma, D8001) and stained with hematoxylin, the sections were captured images by using a laser scanning confocal microscopy (Zeiss) and analyzed with ImageJ software.

### 2.11 Statistical analysis

All quantified data were expressed as means ± SEM from triplicate samples and analyzed by using GraphPad Prism 7.0 software. Shapiro–Wilk test was used to assess data normality. Statistical significance of differences between two groups was determined by Student's *t*‐test, while that among multiple groups was determined by the one‐way analysis of variance (ANOVA). For the various analyses, *p* < 0.05 was considered statistically significant.

## 3 RESULTS

### 3.1 Purification and characterization of RAs cultured in vitro

**FIGURE 1:**
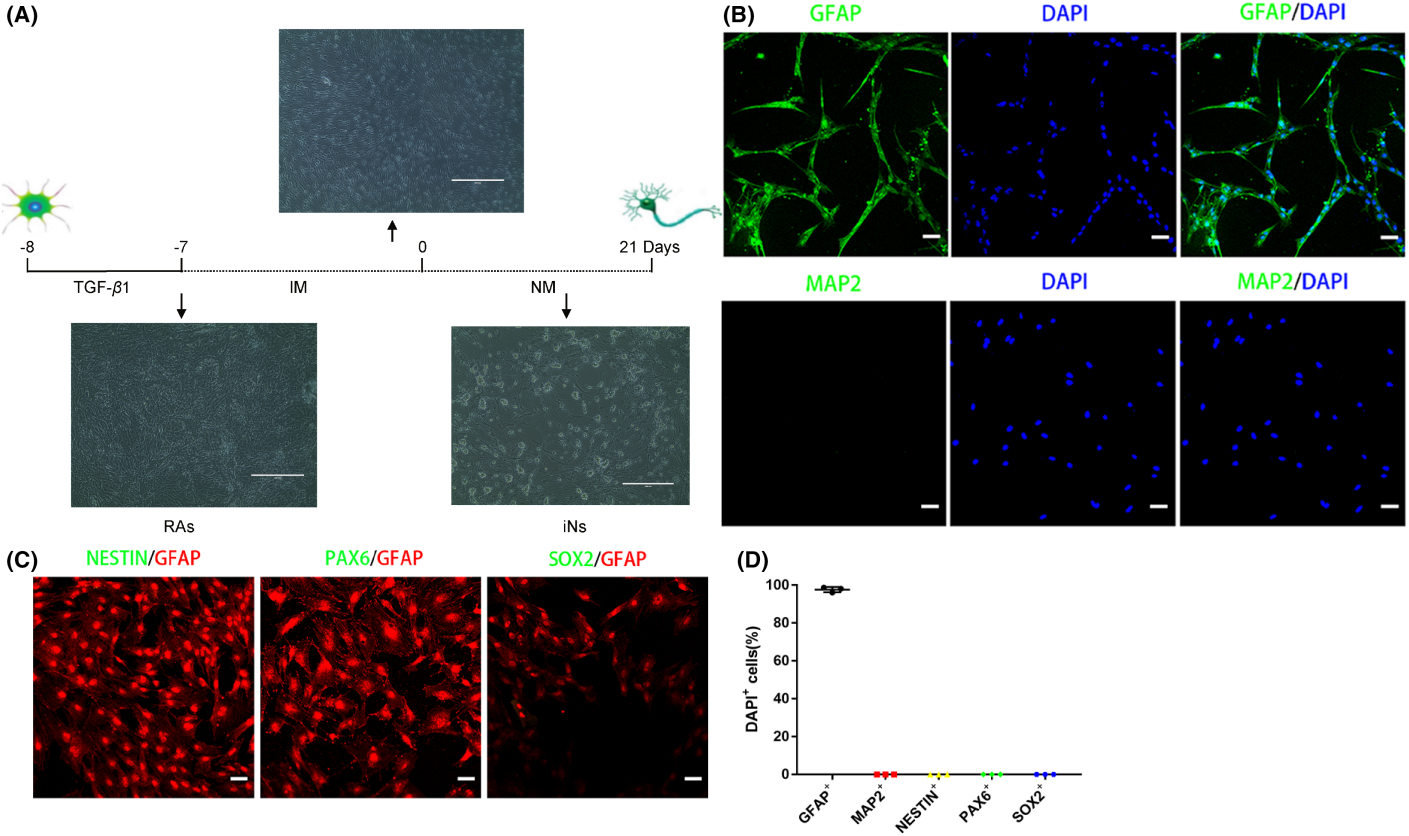
Morphological and biochemical characteristics of primary rat RAs. (A) Schematic drawing showing the neuronal induction protocol and cell morphological changes on different days in vitro. Scale bars, 400 μm. IM, Rg1 induction medium; NM, neuron conditioned medium. (B, C) Immunostaining showing that the majority of cultured cells expressed the RA marker GFAP, but not the neuronal marker MAP2 or the NPCs markers, NESTIN, PAX6, and SOX2. Scale bars, 50 μm. (D) Quantitative analysis of cultured cells for marker expression of astrocytes, neuronal cells, or NPCs. The data were presented as the means ± SEM from three independent cell culture preparations (*n* = 3).

To avoid the presence neural progenitor cells (NPCs), the cultured cells were sequentially treated with differential adhesion, constant temperature oscillation overnight and serial passage to the third generation.^20^ Then, exogenous TGF‐β1 was added to stimulate the cultured cells to become RAs.^22^ When observed under an inverted microscope, the morphology of the cultured cells was typically star‐shaped, polygonal. Many protrusions of them were short and thick (Figure 1A). Indeed, more than 95% of the cultured cells were positive for the RA marker GFAP but negative for NPC markers (NESTIN, PAX6, and SOX2). In addition, the cultured cells did not express neuronal markers MAP2 (Figure 1B–D).

These results suggested that the cultured cells were mainly RAs without detectable contamination of NPCs or neuronal cells.

### 3.2 Direct transdifferentiation of RAs into iNs by ginsenoside Rg1

**FIGURE 2:**
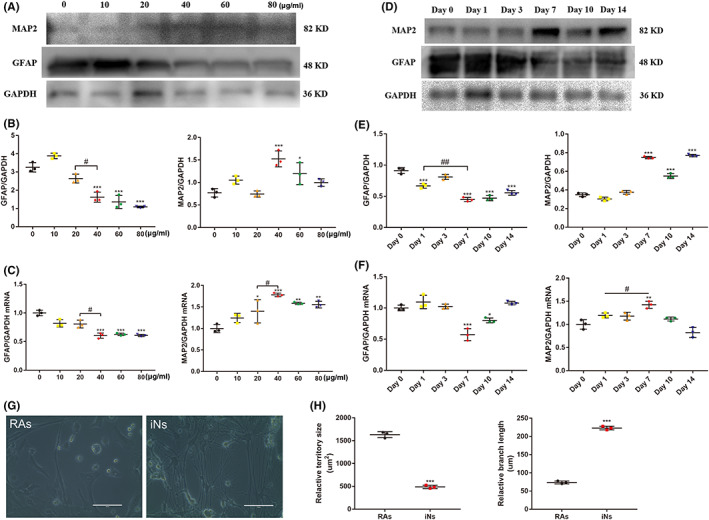
Optimal condition of induction and attenuation of astrocytic characteristics. (A–C) Western blot, densitometric quantitation, and qRT‐PCR analysis of GFAP and MAP2 in iNs with different concentrations of ginsenoside Rg1. The data were presented as the means ± SEM from three independent cell culture preparations (*n* = 3). *p < 0.05, **p < 0.01 and ***p < 0.001 versus the Day 0 group; ^##^ *p* < 0.01 versus the Day 1 group. (D–F) Western blot, Densitometric quantitation and qRT‐PCR analysis of GFAP and MAP2 in iNs on different days during ginsenoside Rg1 induction. All protein expression levels and signals were normalized to GAPDH. The data were presented as the means ± SEM from three independent cell culture preparations (*n* = 3). *p < 0.05, **p < 0.01 and ***p < 0.001 versus 0 group; ^#^ *p* < 0.05 versus 20 group. (G) Phase‐contrast image showing the changes from stellate shape to neuronal morphology after ginsenoside Rg1 induction. Scale bars, 100 μm. (H) Quantitative analysis of the total branch length and total territory size, defining as the two‐dimensional that was measured by the border of the cell body, of iNs and RAs. The data were presented as the means ± SEM from three independent cell culture preparations (*n* = 3). ***p < 0.001 versus the RAs group

It has been demonstrated that RAs, serving as one of the acting cells in CNS after SCI, could be induced to the cells with some characteristics of neurons.^15^, ^23^ Ginsenoside Rg1 has also been proved with several neuroprotective effects.^24^ Therefore, we selected ginsenoside Rg1 as the pivotal monomeric drug inducing RAs into iNs. Taking previous studies as a reference, 40 μg/ml was the best concentration of ginsenoside Rg1 when utilized to regulate the function of RAs.^20^, ^21^ To identify the optimal transdifferentiation efficiency corresponding to the concentration of ginsenoside Rg1, we set a specific concentration gradient to verify the optional concentration. As expected, 40 μg/ml was the most appropriate concentration according to mRNA levels of *GFAP* and *MAP2*, which was consistent with the expression of their protein (Figure 2A–C). In order to figure out the optimal induction day, we prolonged the induction until indicated days. Western blot results at indicated days of the induction demonstrated that the optimal number induction days was 7. Additionally, the qRT‐PCR analysis of different induction days showed the same result (Figure 2D–F). On day 7 after ginsenoside Rg1 induction, the morphology of RAs experienced a quickly change from a flat, polygonal star shape into an irregular polar shape. After continuous induction until day 7 and the replacement of medium with neuron maturation medium containing forskolin and neurotrophic factors (BDNF, GDNF, and NT3), the induced cell bodies had smaller territory sizes and became more compact, with more complex and slenderer neuron‐like branching structures. In contrast, RAs in the control group without ginsenoside Rg1 had no significant morphological change (Figure 2G,H).

**FIGURE 3:**
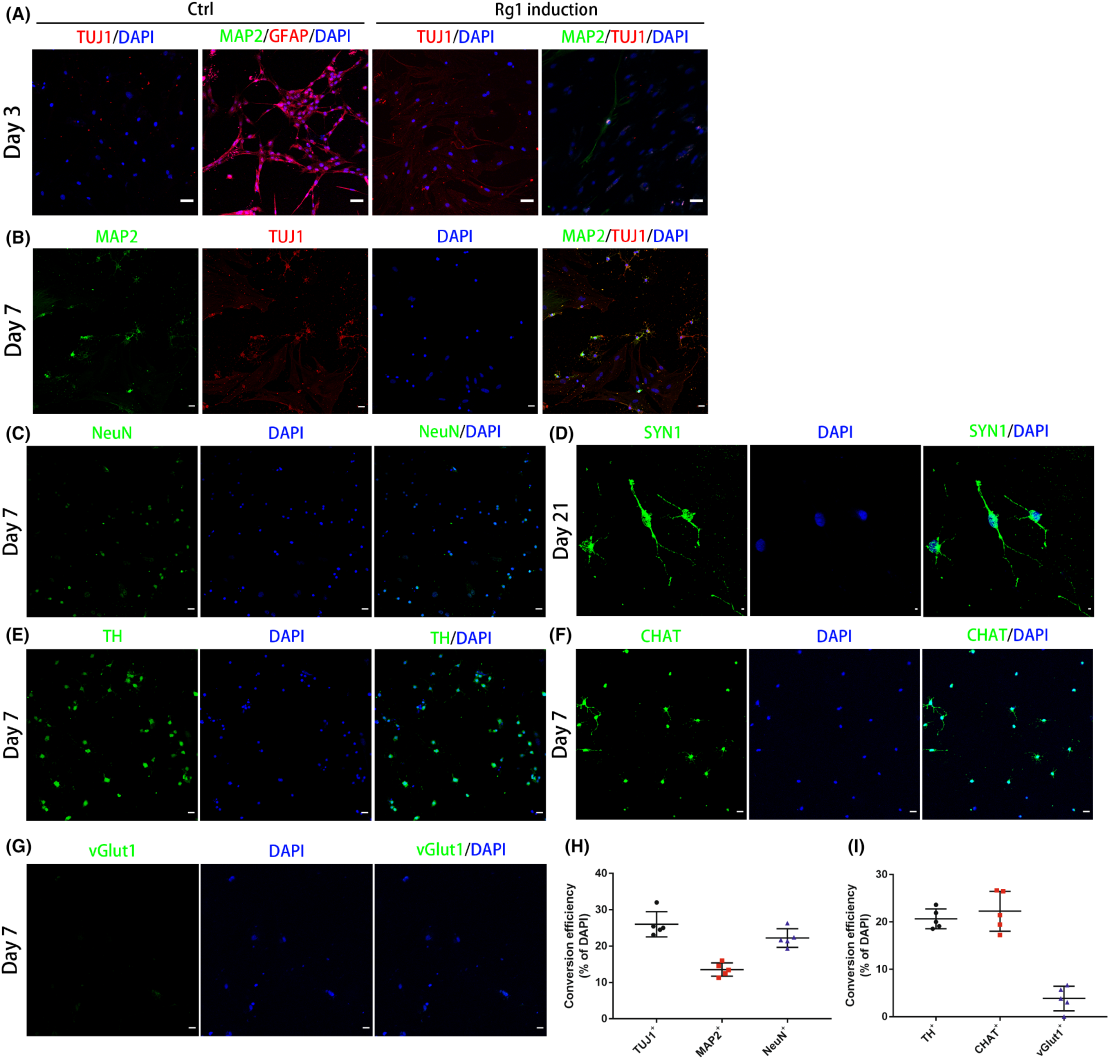
Transdifferentiation of RAs into iNs by ginsenoside Rg1. (A) Expression of TUJ1, MAP2, and GFAP in the control group and ginsenoside Rg1 induction group on day 3. Scale bars, 50 μm. (B–D) Immunostaining showing that iNs were positive for TUJ1, MAP2, and NeuN on day 7 (Scale bars, 50 μm) and SYN1 on day 21 (Scale bars, 20 μm). (E–G) Immunostaining of different subtype markers revealed that iNs were more like cholinergic (CHAT) and dopaminergic (TH) neurons but rarely glutamatergic (vGlut1) neurons. Scale bars, 50 μm. (H, I) Quantitative analysis of neuronal conversion efficiency and neuronal subtypes were performed. The data were presented as the means ± SEM from five independent cell culture preparations (*n* = 5).

Immunostaining results showed that iNs were positive for early neuronal marker TUJ1 after 3 days of the induction, and some of these iNs were positive for mature neuronal marker MAP2. In the control group, the cells were negative for TUJ1 or MAP2 (Figure 3A). To determine the neuronal conversion efficiency and neuronal subtypes, we calculated the percentages of TUJ1, MAP2, NeuN, TH, CHAT, and vGlut1‐positive cells with neuron‐like cell morphologies relative to the total DAPI^+^ cells on day 7 (Figure 3B,C,E–G). Quantitative analysis showed that the conversion efficiencies for TUJ1, MAP2, and NeuN were approximately 26.0 ± 1.5%, 13.6 ± 0.8%, and 22.2 ± 1.1%, respectively. As for neuronal subtypes, iNs were predominately CHAT^+^ (20.6 ± 0.9%) cholinergic and TH^+^ (22.3 ± 1.9%) dopaminergic, but rarely vGlut1^+^ (3.9 ± 1.2%) glutamatergic neurons (Figure 3H,I). Moreover, we found that iNs were immunopositive for the neurosynaptic junction marker SYN1 on day 21 after the induction (Figure 3D).

These results indicated that the best transdifferentiation efficiency was with 40 μg/ml ginsenoside Rg1 for 7 days and RAs could indeed acquire a neuronal fate through promoting the expression of neuronal markers and blocking that of RA markers, mainly forming cholinergic and dopaminergic neurons.

### 3.3 Transdifferentiation of iNs without a neural progenitor stage

**FIGURE 4:**
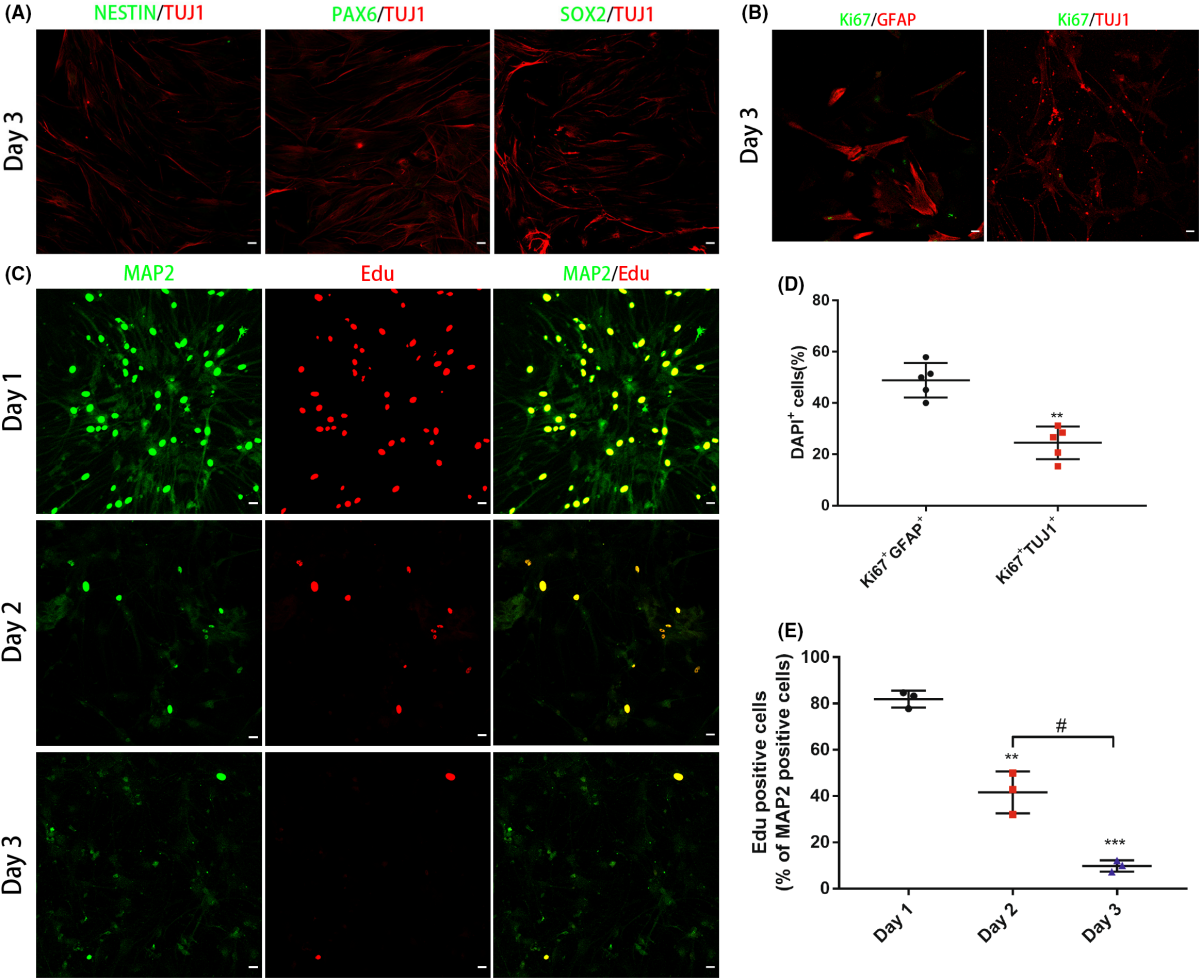
Ginsenoside Rg1 directly transdifferentiated RAs into iNs without passing through a proliferative NPCs stage. (A) Immunostaining showing that iNs were almost negative for the NPC markers NESTIN, PAX6, and SOX2 after ginsenoside Rg1 induction on day 3. Scale bars, 50 μm. (B) Expression of Ki67 after ginsenoside Rg1 induction compared to that observed without the induction on day 3. Scale bars, 50 μm. (C) Immunostaining showing that fewer and fewer MAP2‐positive cells were Edu positive on days 1, 2, and 3 after ginsenoside Rg1 induction. Scale bars, 50 μm. (D) Quantitative analysis of Ki67 positive RAs or iNs on day 3. The data were presented as the means ± SEM from five independent cell culture preparations (*n* = 5). **p < 0.01 versus the RAs group. (E) Quantitative analysis of Edu positive iNs on day 1, 2, and 3 after ginsenoside Rg1 induction. The data were presented as the means ± SEM from three independent cell culture preparations (*n* = 3). **p < 0.01 and ***p < 0.001 versus the Day 1 group; ^#^ *p* < 0.05 versus the Day 2 group

To explore whether RAs could be transdifferentiated into NPCs during ginsenoside Rg1 induction, we detected the expression of the NPC markers NESTIN, PAX6, and SOX2 and proliferation marker Ki67 in iNs. There was almost no expression of NESTIN, PAX6, or SOX2 on day 3 of induction according to the results of immunostaining. Meanwhile, the morphological changes of iNs that promoted by ginsenoside Rg1 induction were not similar to the morphology of NPC‐like cells, which was characterized by the formation of cell clusters (Figure 4A). The number of Ki67^+^ cells was significantly decreased on day 3 after induction compared with the number in the control group (Figure 4B,D). We further performed an Edu incorporation experiment in iNs on day 1, 2, and 3. The results showed that little or no Edu incorporation was observed on day 2 or 3, indicating that there was no proliferation occurred during ginsenoside Rg1 induction (Figure 4C,E).

These results suggested that RAs were transdifferentiated into iNs directly without passing through the stage of NPCs.

### 3.4 Protein and gene expression of iNs were closer to that of neurons than RAs

**FIGURE 5:**
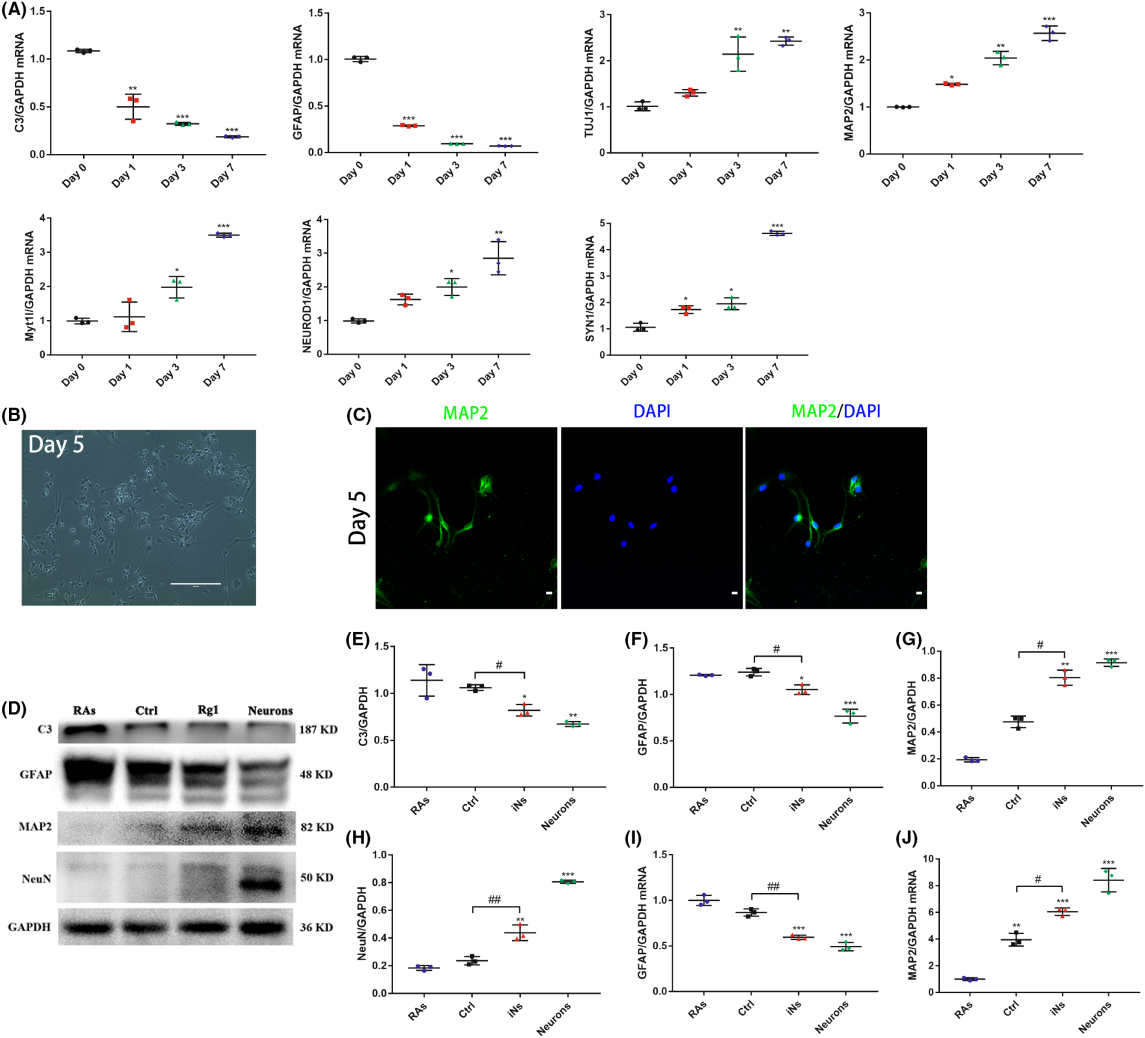
Gene expression profiles of iNs were closer to neurons. (A) qRT‐PCR analysis of mRNA expression levels of gene *C3*, *GFAP*, *TUJ1*, *MAP2*, *Myt1l*, *NEUROD1*, and *SYN1* during ginsenoside Rg1 induction. The data were presented as the means ± SEM from three independent cell culture preparations (*n* = 3). *p < 0.05, **p < 0.01 and ***p < 0.001 versus the Day 0 group. (B, C) Phase‐contrast image of rat neurons with typical neuronal morphology on day 5 (Scale bars, 200 μm) and immunostaining showing that cultured rat neurons expressed the neuronal marker MAP2 (Scale bars, 20 μm). (D–J) Densitometric quantitation and mRNA expression of C3, GFAP, MAP2, and NeuN in RAs, Ctrl, iNs, and neurons on day 7 after ginsenoside Rg1 induction according to western blot and qRT‐PCR analysis. All protein expression levels and signals were normalized to GAPDH. The data were presented as the means ± SEM from three independent cell culture preparations (*n* = 3). *p < 0.05, **p < 0.01 and ***p < 0.001 versus the RAs group; ^#^ *p* < 0.05 and ^##^ *p* < 0.01 versus the control group

We used qRT‐PCR to figure out the changes of specific gene expression of iNs during ginsenoside Rg1 induction. The levels of mRNA and the protein expression of RA markers *C3* and *GFAP* were downregulated, while those of neuronal markers *TUJ1*, *MAP2*, and *SYN1* were upregulated during the induction. Moreover, the proneuronal transcription factor‐encoding genes *NEUROD1* and *Myt1l* were upregulated after the induction (Figure 5A).

In order to confirm whether iNs were closer to neurons than RAs, we first cultured the rat neurons and maintained them in a defined, serum‐free NM for 5 days. The primary neuronal cells were demonstrated by immunostaining of neuronal markers MAP2. Most cultured cells were positive for MAP2. Meanwhile, the cultured cells also showed typical neuronal morphology with MAP2^+^ axons (Figure 5B,C). Then, we performed western blot to detect the protein expression of RA and neuronal markers in RAs, Ctrl, iNs, and neurons on day 7 after the induction (Figure 5D). The results revealed that the protein expression of iNs was closer to those of neurons (Figure 5E–H). Consistently, the mRNA levels of *GFAP* and *MAP2* were similar to the protein expression of those genes (Figure 5I,J).

These results suggested that RAs inducted by ginsenoside Rg1 adopted a neuronal identity and inhibited astrocytic expression.

### 3.5 Continuous culturing and apoptotic ability after ginsenoside Rg1 induction

**FIGURE 6:**
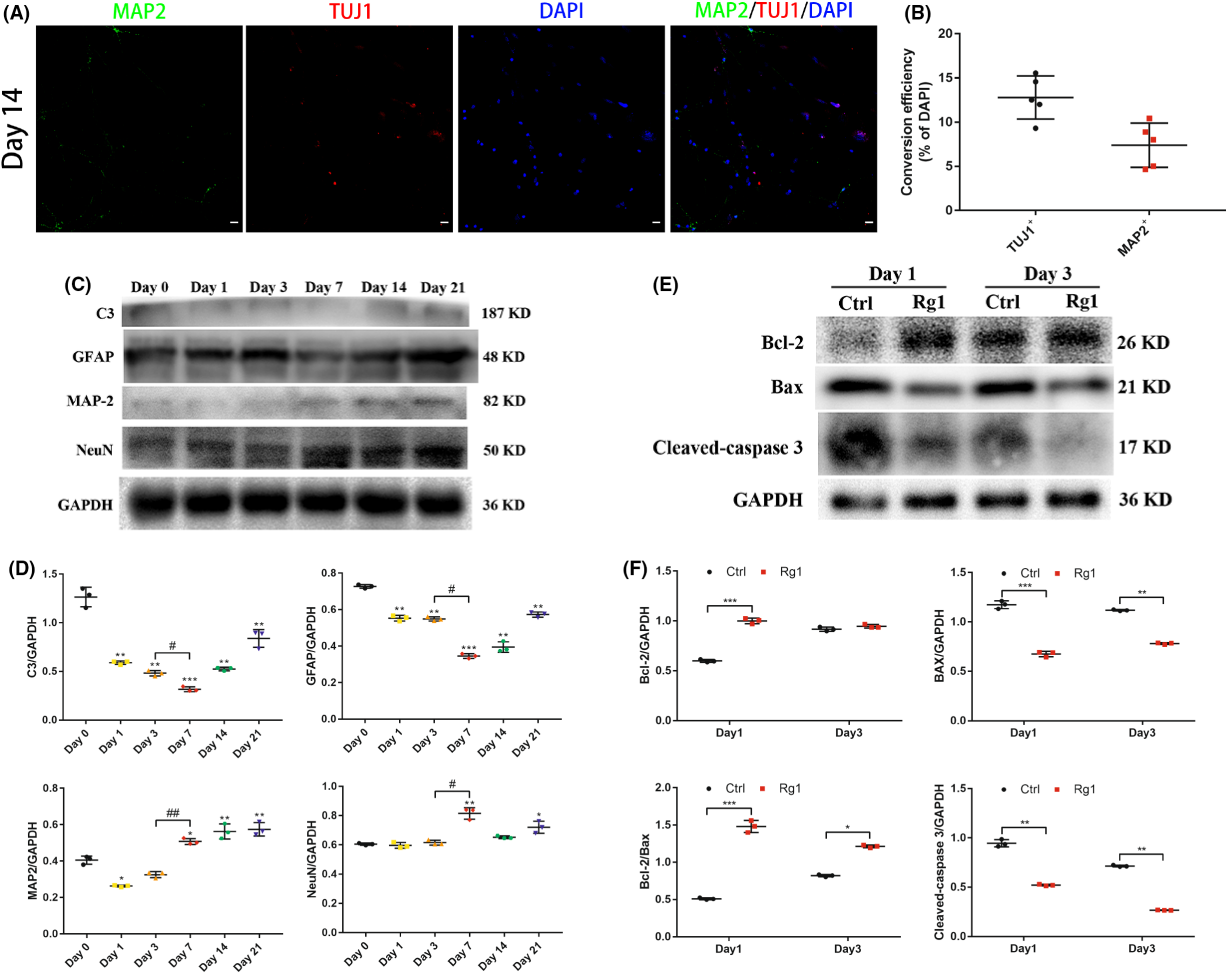
Influence of prolonged culturing time on iNs. (A) Immunostaining showing that iNs were positive for TUJ1, MAP2 on day 14. Scale bars, 50 μm. (B) Quantitative analysis of neuronal conversion efficiency was performed. The data were presented as the means ± SEM from five independent cell culture preparations (*n* = 5). (C, D) Western blot and densitometric quantitation of C3, GFAP, MAP2, and NeuN in iNs on different days after ginsenoside Rg1 induction. The data were presented as the means ± SEM from three independent cell culture preparations (*n* = 3). *p < 0.05, **p < 0.01 and ***p < 0.001 versus the Day 0 group; ^#^ *p* < 0.05 and ^##^ *p* < 0.01 versus the Day 3 group. (E, F) Western blot and densitometric quantitation of apoptotis‐related protein in the control group and ginsenoside Rg1 induction group on day 1 and 3 after ginsenoside Rg1 induction. All protein expression levels were normalized to GAPDH. The data were presented as the means ± SEM from three independent cell culture preparations (*n* = 3). *p < 0.05, **p < 0.01 and ***p < 0.001 versus the control group

To figure out whether continuous culturing would affect the transdifferentiation efficiency and related characteristics in iNs, we prolonged culturing time until day 21. Immunostaining results and quantitative analysis showed that the conversion efficiencies on day 14 resembled that on day 7 (Figure 6A,B). Meanwhile, there was no more encouragement in neuronal expression or inhibitory in astrocytic expression in iNs according to western blot (Figure 6C,D).

Additionally, we verified the apoptotic ability of iNs by utilizing western blot on day 1 and 3 after the induction (Figure 6E). The results revealed that expression levels of the proapoptotic protein Bax and cleaved‐caspase 3 in iNs were significantly increased compared with those in the control group, while expression of the antiapoptosis protein Bcl‐2 and ratio of Bcl‐2 to Bax was downregulated (Figure 6F).

These results suggested that prolonged culturing time was no use to neuronal transdifferentiation and the antiapoptotic ability of them was improved obviously.

### 3.6 Promotion of functional recovery and reduction of lesion cavity volume with ginsenoside Rg1 treatment after SCI

**FIGURE 7:**
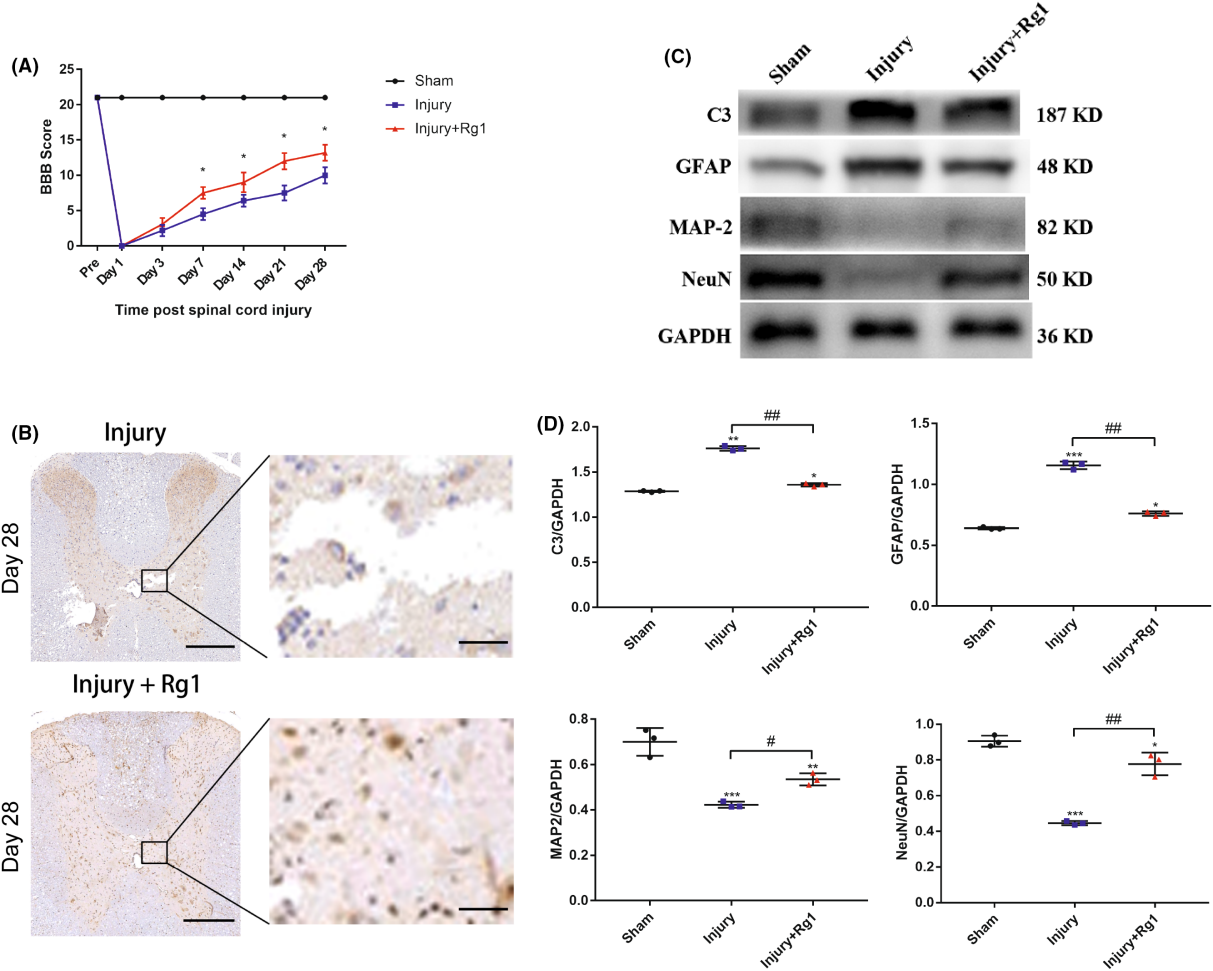
Promotion of functional recovery and regeneration of neurons in vivo. (A) The BBB score was performed to evaluate motor function at indicated times after SCI in the sham group, the injury group, and Rg1 treatment group. The data were presented as the means ± SEM from 10 animals (*n* = 10). *p < 0.05 versus the injury group. (B) Immunohistochemical staining of MAP2 of coronal sections in the injury group and Rg1 treatment group on day 28 post SCI. Upper scale bars, 200 μm. Lower scale bars, 100 μm. (C, D) Western blot and densitometric quantitation of C3, GFAP, MAP2, and NeuN in the sham group, the injury group, and the Rg1 treatment group on day 7 post SCI All protein expression levels were normalized to GAPDH. The data were presented as the means ± SEM from spinal tissue of three animals (*n* = 3). *p < 0.05, **p < 0.01 and ***p < 0.001 versus the sham group; ^#^ *p* < 0.05 and ^##^ *p* < 0.01 versus the injury group

To assess motor function recovery of different groups, a modified BBB scale was used to measure the locomotive function of rats on the indicated day post SCI. There was no detectable deficiency in locomotive movements of all rats before the operation according to the BBB scores. There was also no deficiency detected in locomotive movements of the rats from the sham group after the operation, suggesting that the laminectomy itself had no damage on locomotive ability. On day 1 and 3 post SCI, the rats from two other groups went through severe damage on locomotive ability, exhibiting complete bilateral paralysis (BBB score = 0). However, the rats in Rg1 treatment group exhibited significant improvement in the BBB scores compared with the rats in vehicle treatment group from the first week post SCI until sacrifice in the fourth week (Figure 7A). We next investigated the effect ginsenoside Rg1 on tissue repair after SCI by using immuohistochemistry staining to detect the expression of neuronal marker MAP2 (Figure 7B). The results showed that the lesion cavity volume was significantly reduced in the rats of Rg1 treatment group on day 28 post SCI. Meanwhile, the expression of MAP2 in Rg1 treatment group was significantly higher compared with that in the injury group. We further performed western blot to detect the protein expression of C3, GFAP, MAP2, and NeuN at the spinal lesion site on day 28 post SCI (Figure 7C). As expected, the protein expression of RA markers C3 and GFAP was significantly downregulated while that of neuronal markers MAP2 and NeuN were significantly upregulated (Figure 7D).

These results indicated that ginsenoside Rg1 treatment promoted functional recovery and reduced the spinal cord injury lesion cavity volume efficiently through encouraging neuronal expression and inhibiting astrocytic expression in vivo.

### 3.7 Direct transdifferentiation of RAs into iNs with ginsenoside Rg1 by the suppression of Notch/Stat3 signal pathway

**FIGURE 8:**
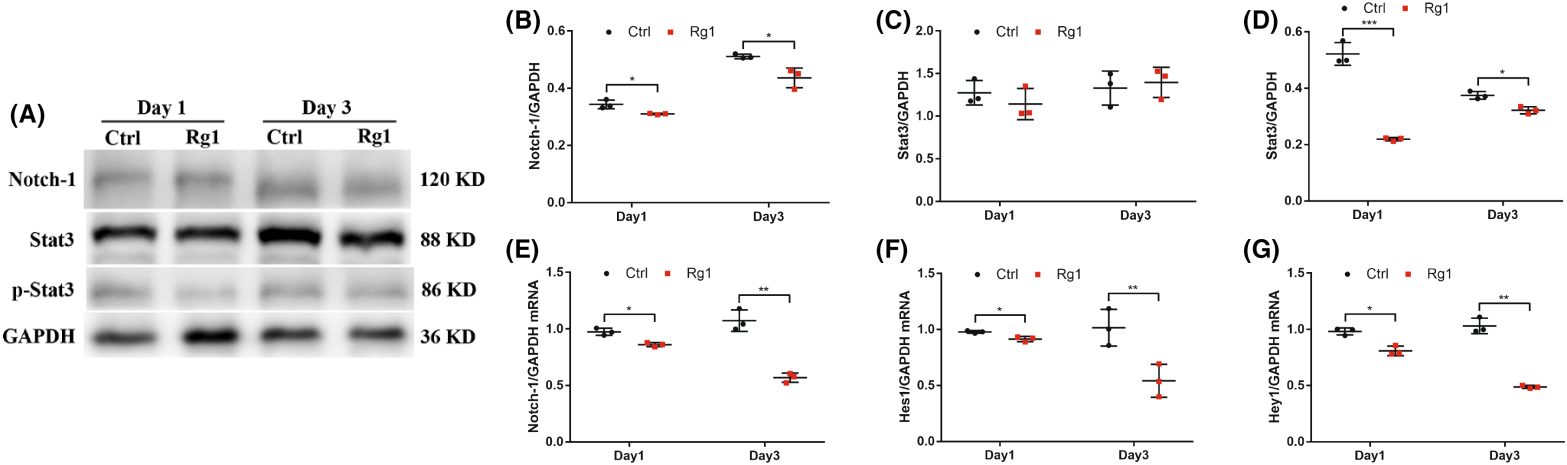
Expression profiles of components of the Notch/Stat3 signal pathway in iNs induced by ginsenoside Rg1. (A–D) Western blot and densitometric quantitation of Notch‐related protein in the control group and ginsenoside Rg1 induction group on day 1 and 3 after ginsenoside Rg1 induction. All protein expression levels were normalized to GAPDH. The data were presented as the means ± SEM from three independent cell culture preparations (*n* = 3). *p < 0.05 and ***p < 0.001 versus the control group. (E–G) qRT‐PCR analysis of mRNA expression levels of *Notch‐1*, *Hes1*, and *Hey1* on day 1 and 3 after ginsenoside Rg1 induction. The data were presented as the means ± SEM from three independent cell culture preparations (*n* = 3). *p < 0.05 and **p < 0.01 versus the control group

The Notch pathway plays an important part in cell transformation, proliferation, and apoptosis.^25^ Signal transducer and activator of transcription 3 (Stat3), related to Notch strongly, is also a vital transcription factor to the regulation of neuronal differentiation and functional recovery with SCI.^26^, ^27^ Accordingly, we initially examine the levels of protein in Notch/Stat3 signal pathway to confirm the effect of ginsenoside Rg1 induction (Figure 8A). The results showed that there was an significant reduction of Notch‐1 and p‐Stat3 on day 3 after gisenoside Rg1 induction (Figure 8B–D). In the meanwhile, to provide further evidence for the change of mRNA levels in Notch/Stat3 signal pathway, we detected some downstream genes such as *Notch‐1*, *hairy and enhancer of split 1* (*Hes1*), and *Hey1*, and the results were consistent with expected (Figure 8E–G).

These results suggesting that there was an inhibitory action of ginsenoside Rg1 on Notch/Stat3 signal pathway and an enhancement on neuronal transdifferentiation.

## 4 DISCUSSION

Following the primary injury caused by immediate impact after SCI, delayed injury via excessive neuroinflammatory response leads to severer damage.^2^, ^28^ Stimulated by inflammatory factors, astrocytes will lose their original function and contribute to the proliferation of gliar scars.^4^, ^5^ Taking astrocytes' proximity in lineage to neurons into account, the directly transdifferentiation of RA‐to‐neuron has become a burgeoning therapy.^29^, ^30^ Compared to direct lineage conversion achieved by viral intervention, transdifferentiation achieved by the mixed small molecules is more practical and reliable.^31^, ^32^ In recent studies, several defined small molecules had been utilized to induct neuronal transdifferentiation.^8^, ^9^ However, more molecules that promote the transdifferentiation used during the therapy, more uncontrollable the curative effect will possibly be. Ginsenoside Rg1 has been proven to be effective firmly in influencing several degeneration diseases including cardiocerebral vascular system, nervous system, and immune system.^17^, ^18^ In our previous studies, we have confirmed that ginsenoside Rg1 treatment could modulate endogenous repair mechanisms after SCI. For example, the migration of olfactory ensheathing cells (OECs) was promoted, which owned the ability of stimulating neuronal regeneration via the PI3K/Akt pathway.^21^, ^33^ Moreover, sorts of neurotrophic factors such as BDNF, GDNF, and nerve growth factor (NGF) as well as cell adhesion factors such as laminin (LN) and fibronection (FN) secreted by RAs increased after the therapy with ginsenoside Rg1.^20^ Therefore, we studied ginsenoside Rg1 induction on RA‐to‐neuron to prove if this promising therapy could be translated into clinical applications.

The transdifferentiation process does not experience an intermediate stage of NPCs. In this study, the expression of NPC markers NESTIN, PAX6, and SOX2 was undetectable throughout ginsenoside Rg1 induction, which was coincident with the results of neuronal conversion induced by small molecules in recent studies.^14^, ^15^ Lack of Edu incorporation into TUJ1 co‐expression after the induction also confirmed that the direct transdifferentiation bypassed the proliferative stage. Given that ginsenoside Rg1 has been proven to be effective in the amelioration of SCI in our previous studies, we further demonstrated the exact effect of ginsenoside Rg1 on the transdifferentiation of RA‐to‐neuron, presenting as the downregulation of RA markers GFAP and C3 and the upregulation of neuronal markers *NEUROD1*, *Myt1l*, TUJ1, MAP2, NeuN, and SYN1. Throughout the transdifferentiation process, *NEUROD1* and *Myt1l* had played significant roles as transcription factors related to directly neuronal conversion.^34^, ^35^ The primary subtypes of iNs were closer to CHAT^+^ cholinergic and TH^+^ dopaminergic neurons, which was coincidence with some research results on the promotion of Alzheimer's disease and Parkinson's disease with ginsenoside Rg1 treatment.^36^, ^37^ Interestingly, C3 has been confirmed overexpressed in neurotoxic A1 astrocytes according to the description of two distinct reactive phenotype astrocytes in many studies.^38^, ^39^ Therefore, the effects that ginsenoside Rg1 has on RAs may be concerned with the transformation between A1 and A2 astrocytes simultaneously.

Neurodegeneration through apoptosis and blocking neurocircuit normal function through stunting axonal growth after SCI must be restricted during the therapeutic window.^24^, ^40^ Secondary neuroinflammatory degeneration contributed to neuronal apoptosis, a programmed cell death, as evidenced by upregulating the protein expression of pro‐apoptotic Bax and cleaved‐caspase 3, and downregulating the protein expression of antiapoptotic Bcl‐2. The upregulation of Bax/Bcl‐2 ratio is also a better marker of apoptotic, presenting the balance between the pro‐ and antiapoptotic protein of the Bcl‐2 family. These negative effects were obviously reduced when ginsenoside Rg1 was added to RAs.

Spinal cord injury results in severe motor disabilities through impairing neuronal regeneration and axonal regrowth in the epicenter of lesion.^2^, ^41^ Relying on the establishment of the SCI model with ginsenoside Rg1 treatment, we observed significant shrinkage of the lesion cavity volume and motor function recovery of the hind limb motor following SCI. Moreover, the tissue repair of SCI was displayed as the decreasing expression of RA markers GFAP and C3 and the increasing expression of neuronal markers MAP2 and NeuN. However, the precise mechanism of ginsenoside Rg1 induction on RA‐to‐neuron remained unclear.

The Notch and Stat pathways have multieffects on the processes during the regulation of cell fate.^27^, ^42^ Notch signaling has been proven to be crucial for neurogenesis, gliogenesis, and neuritogenesis.^25^, ^43^ Meanwhile, numerous studies have shown that the Notch pathway also functions in the proliferation of RAs caused by the immune response after SCI.^44^, ^45^ As expected, we initially demonstrated decreasing expression of Notch‐1, the Notch targets *Hes1* and *Hey1* in the injury lesion with ginsenoside Rg1 treatment after SCI. Affected by Notch signaling, Stat3 can be activated and phosphorylated notably. Indeed, the expression of p‐Stat3 was reduced after ginsenoside Rg1 induction (but not total Stat3 expression). Although there have been some studies on Notch signal and Stat pathway with ginsenoside Rg1, the exactly mechanism of the encouragement of RA‐to‐neuron with ginsenoside Rg1 needs more experiments to confirm.

In summary, ginsenoside Rg1 has a positive effect on the directly transdifferentiation of RA‐to‐neuron after SCI in vivo and vitro. What is more, ginsenoside Rg1 also promotes the recovery of rats' motor function. At last, the mechanism of this process may proceed by blocking Notch/Stat3 signal pathway. The intervention of ginsenoside Rg1 could give a novel and optional select to the clinical applications.

## AUTHOR CONTRIBUTIONS

Kelv Shen, Duanrong Wu, and Zhengfeng Lu designed and performed the experiments. Zhengfeng Lu supervised the research. Kelv Shen and Duanrong Wu performed animal breeding. Baihan Sun, Yin Zhu, Hao Wang, Wenjun Zou, and Yuhang Ma performed most of the other experiments. Kelv Shen, Duanrong Wu, and Yuhang Ma analyzed and interpreted data. Kelv Shen and Duanrong Wu prepared manuscript drafts. Kelv Shen, Duanrong Wu, and Zhengfeng Lu edited the paper. All authors read and approved the final manuscript.

## FUNDING INFORMATION

This work was supported by grants from National Natural Science Foundation of China (No. 82074173, 81971164); and the Jiangsu Province Traditional Chinese Medicine Science and Technology Foundation (YB201956).

## CONFLICT OF INTEREST

The authors declare that they have no conflicts of interest.

## Supporting information


Appendix S1

## Data Availability

The data that supports the findings of this study are available in the Supporting Information of this article.
