# Supplementary material for: Ginsenoside Rg1 promotes astrocyte‐to‐neuron transdifferentiation in rat and its possible mechanism
Source: CNS Neurosci Ther. 2022 Nov 9;29(1):256–69. doi: 10.1111/cns.14000 (PMC9804042; doi:10.1111/cns.14000)

Fig. 2

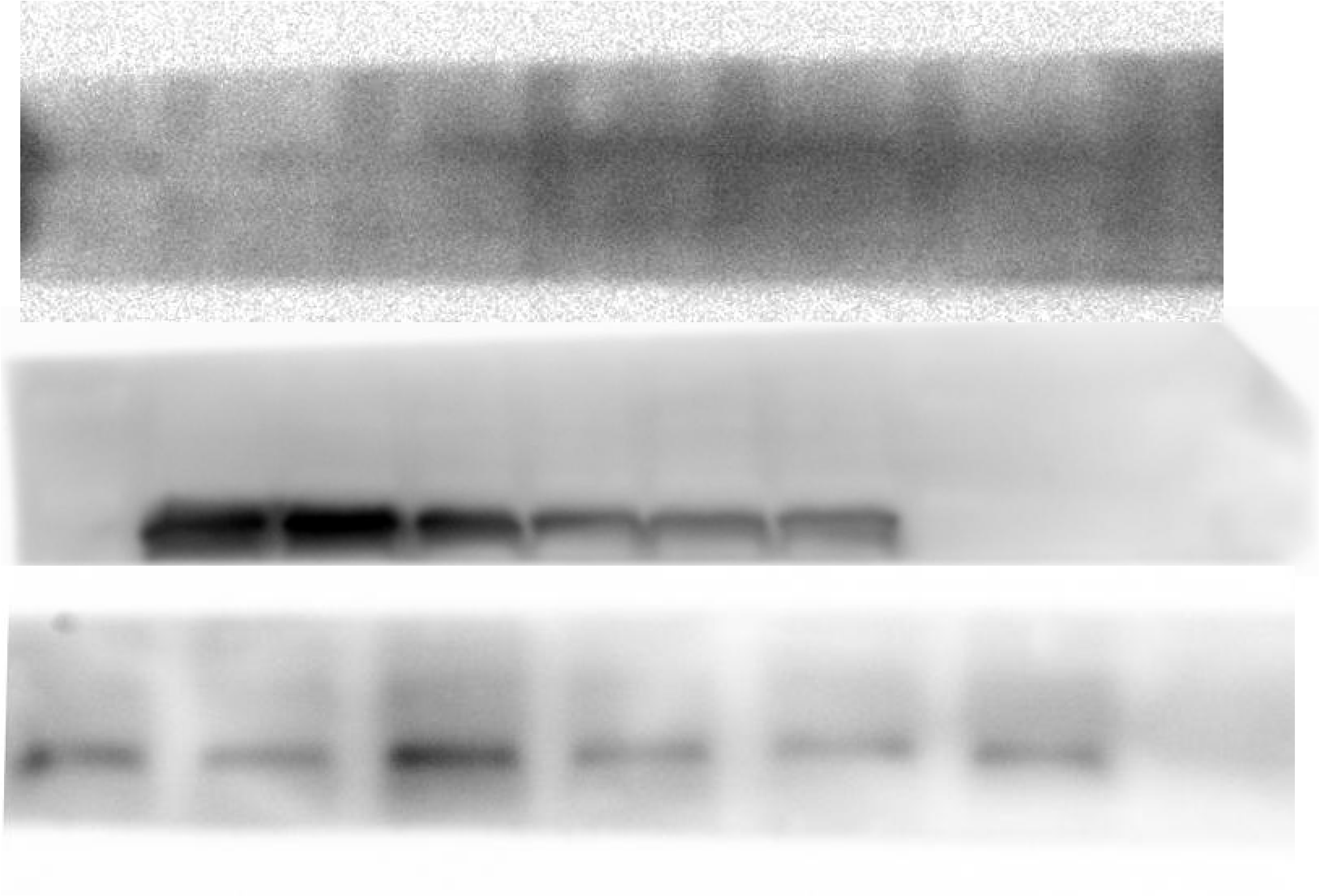

A MAP2

A GFAP

A GAPDH

Fig. 2

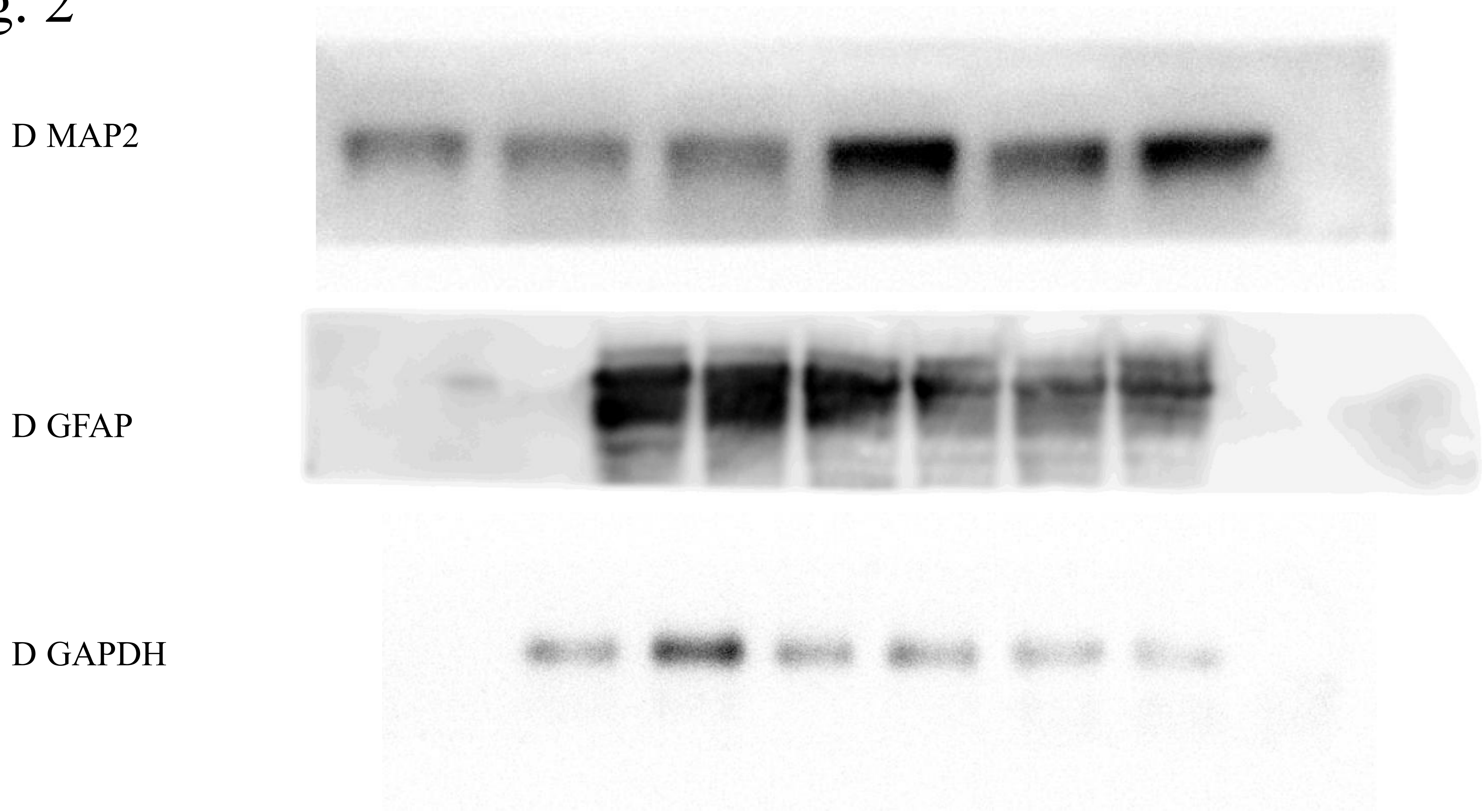

Fig. 5

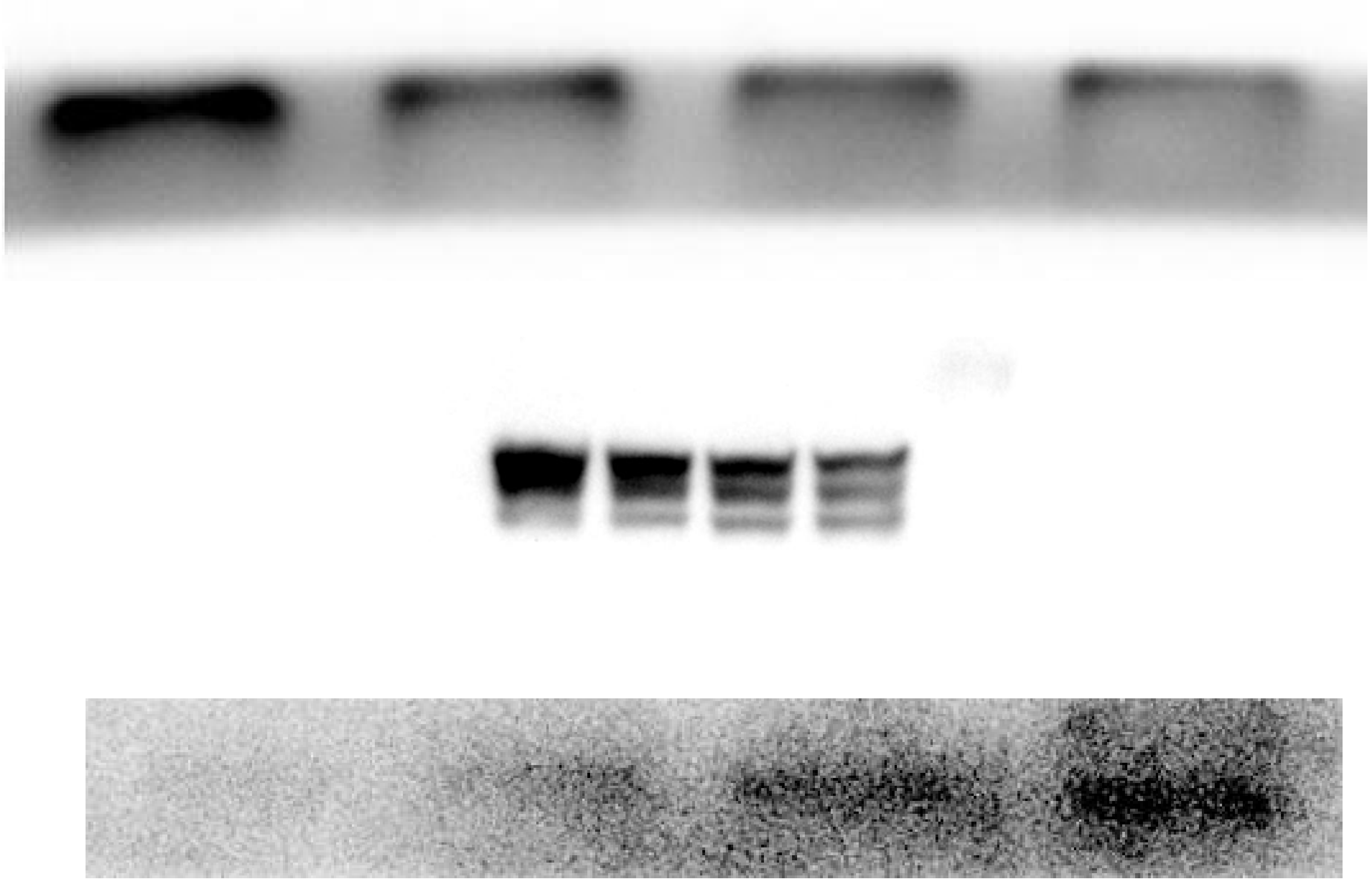

D C3

D GFAP

D MAP2

Fig. 5

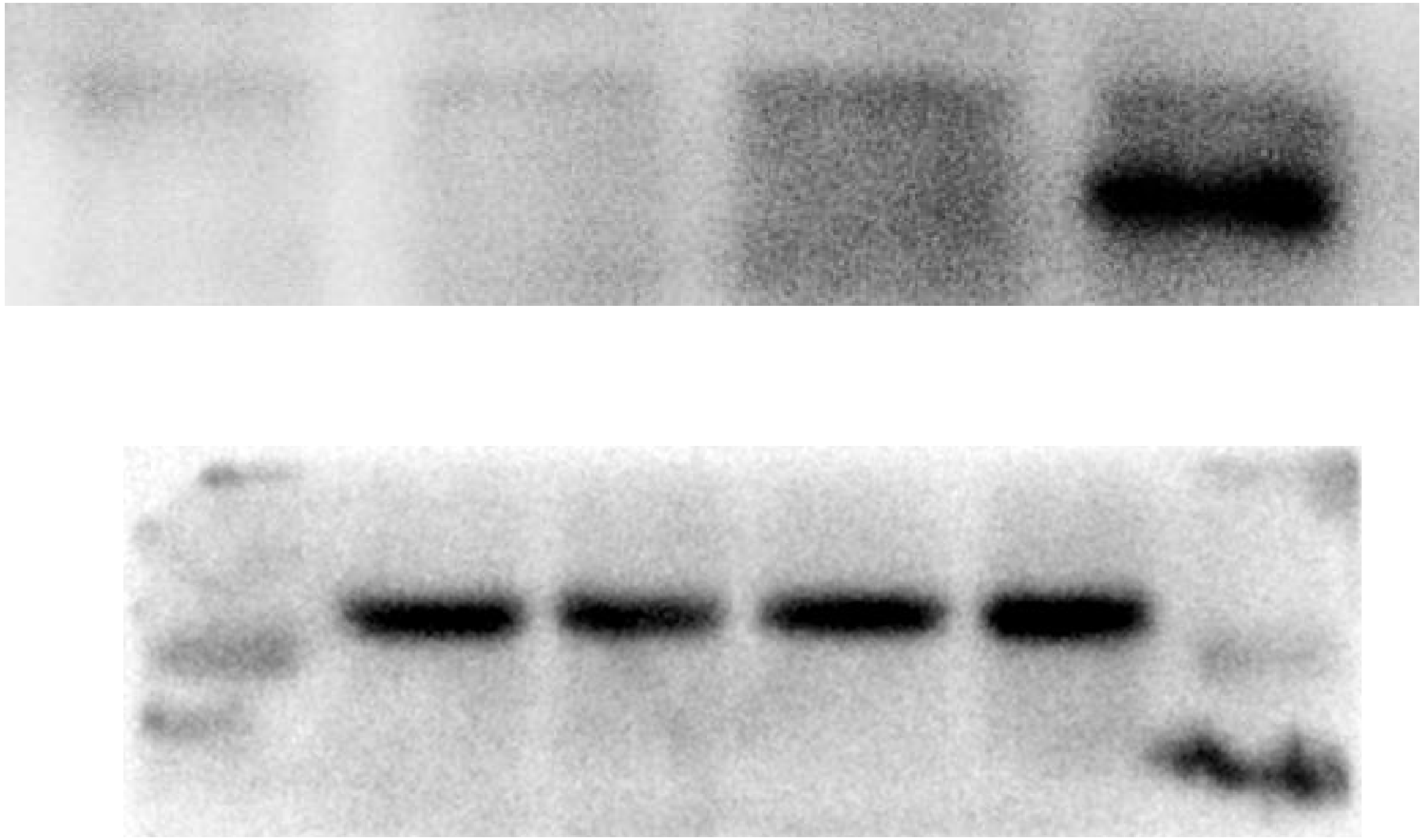

D NeuN

D GAPDH

Fig. 6

C C3

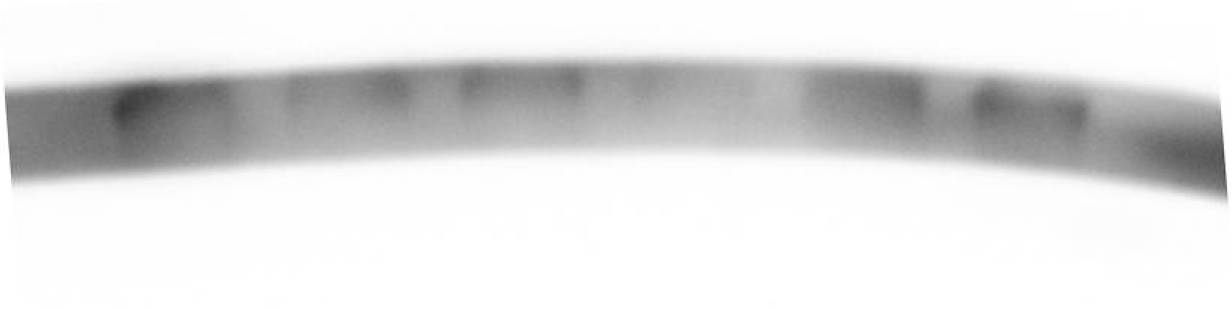

C GFAP

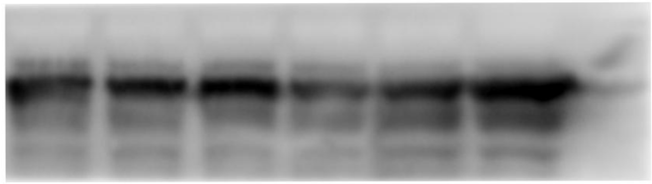

C MAP2

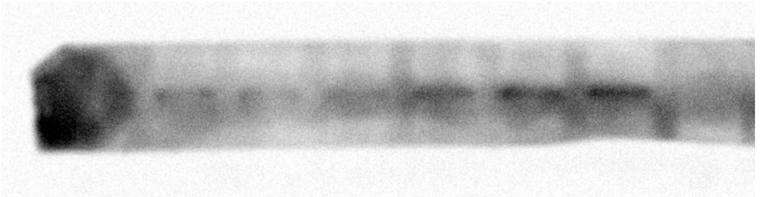

Fig. 6

C NeuN

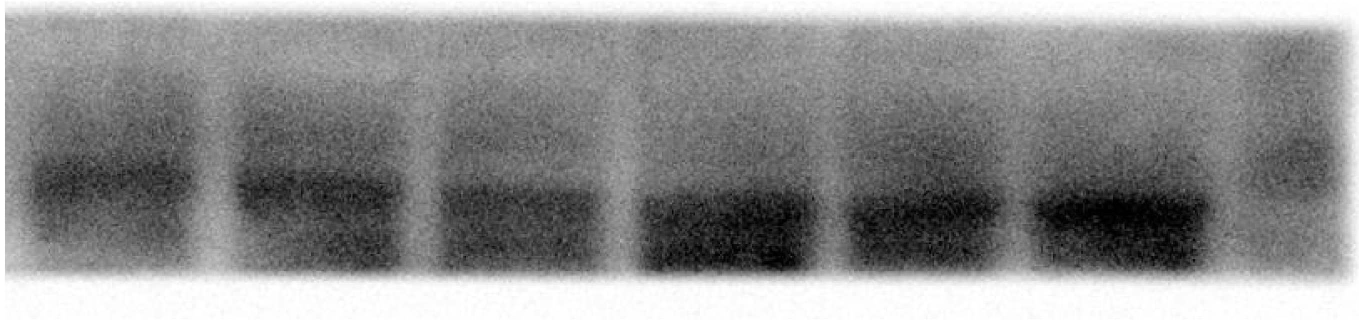

C GAPDH

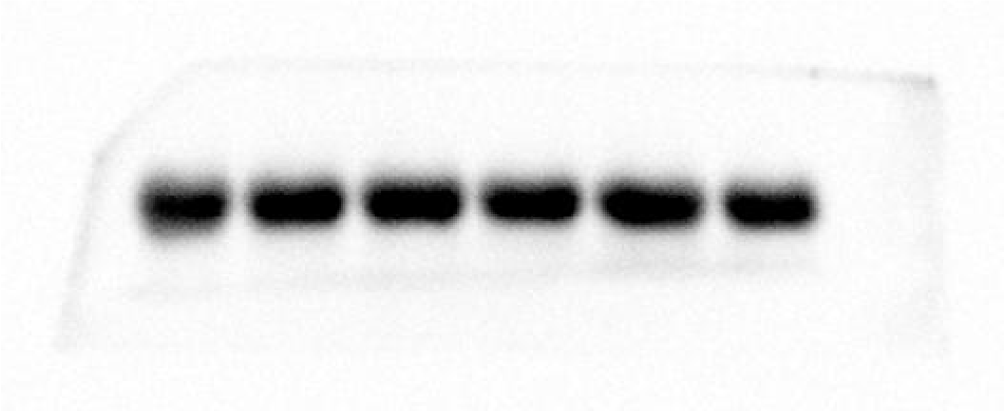

Fig. 6

E Bcl-2

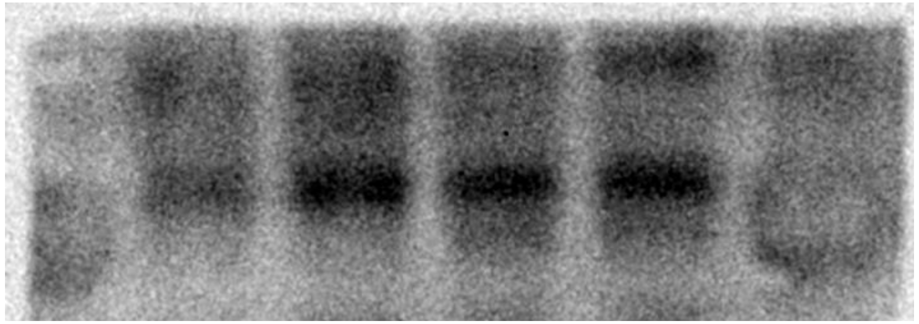

E BAX

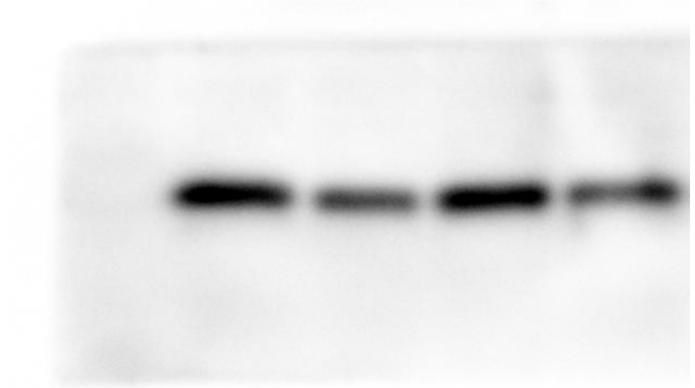

Fig. 6

E Cleaved-caspase 3

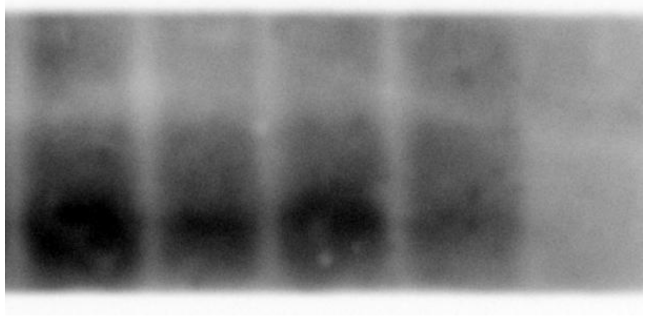

E GAPDH

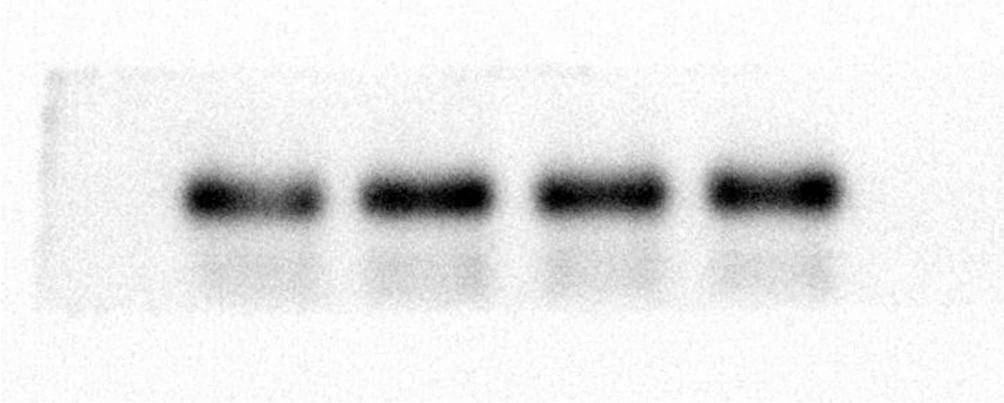

Fig. 7

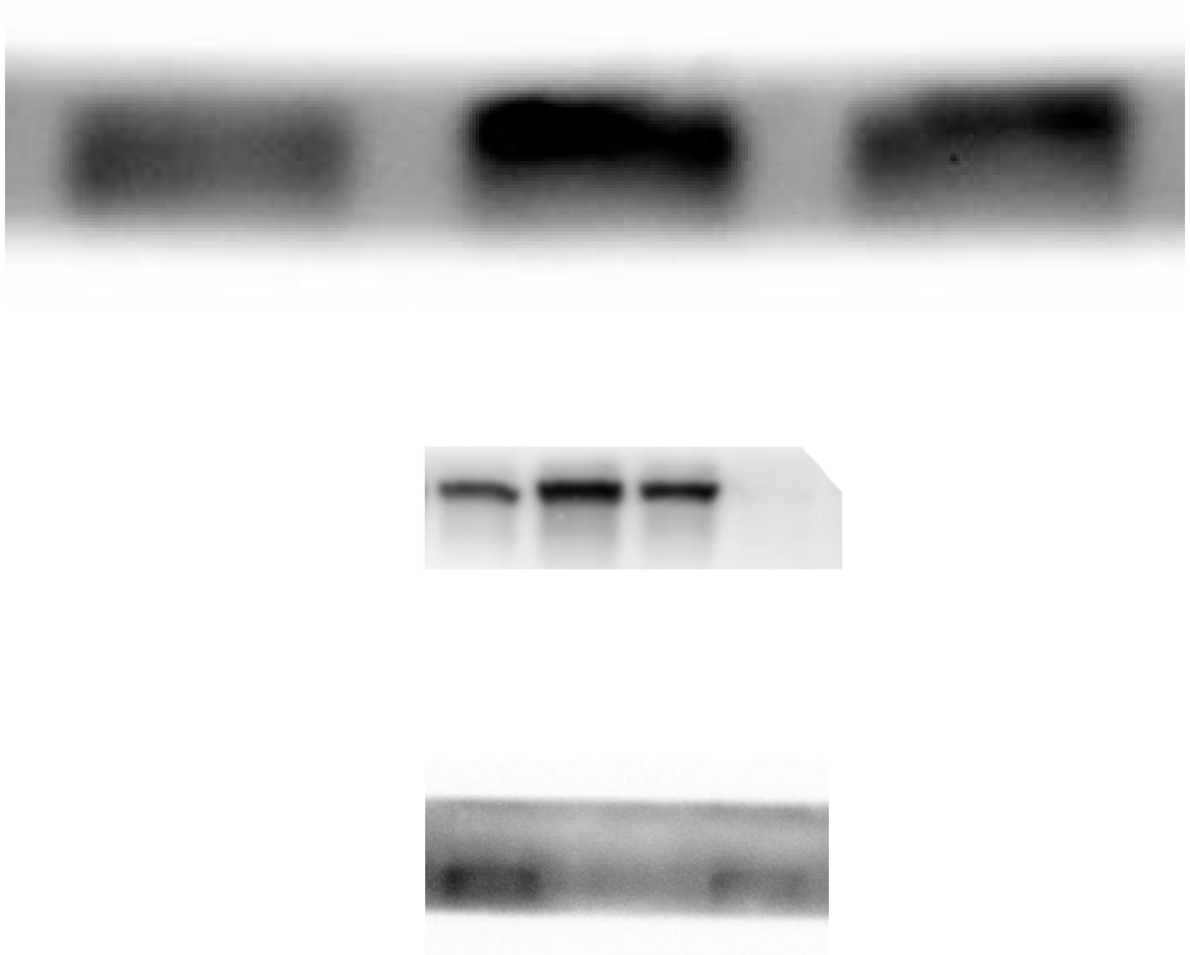

C C3

C GFAP

C MAP2

Fig. 7

C NeuN

C GAPDH

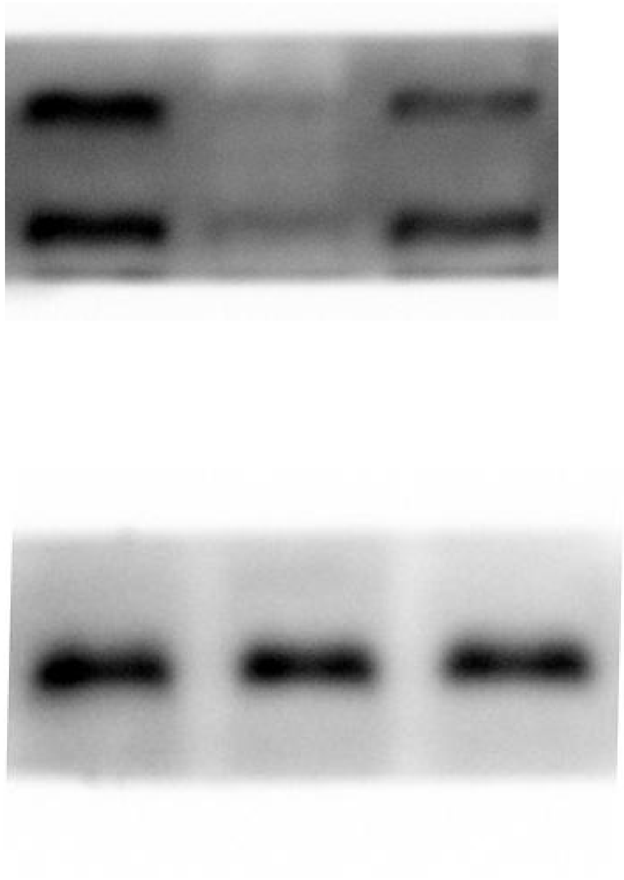

Fig. 8

A Notch-1

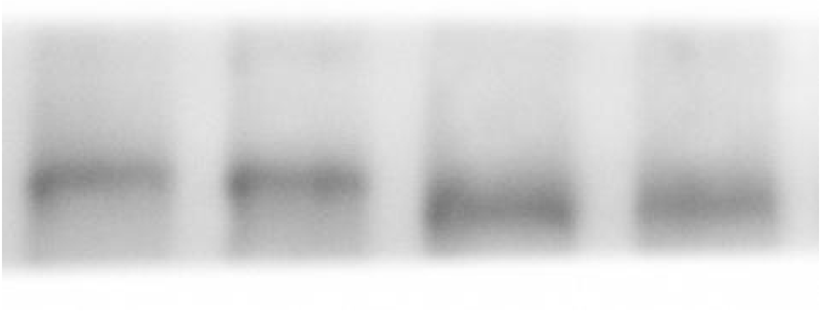

A Stat3

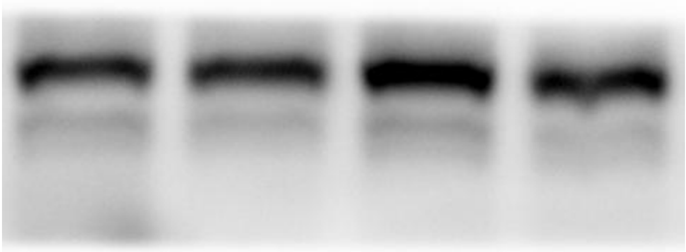

Fig. 8

A p-Stat3

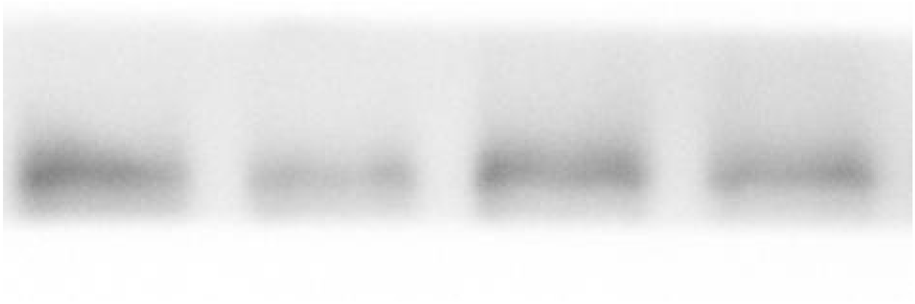

A GAPDH

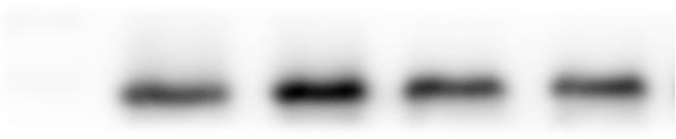

Supplement: Supplementary file 1 — Appendix S1 [file CNS-29-256-s001.pdf]
